# The albumin–bilirubin score is associated with renal decline in diabetic kidney disease: a retrospective study

**DOI:** 10.7717/peerj.21648

**Published:** 2026-08-14

**Authors:** Bailong Cao, Tong Ma, Ying Ban, Yang Zhang, Can Cao

**Affiliations:** Department of Nephrology and Endocrinology, Dongzhimen Hospital, Beijing University of Chinese Medicine, Beijing, China

**Keywords:** Albumin–bilirubin score, Diabetic kidney disease, Estimated glomerular filtration rate, Prognostic biomarker

## Abstract

**Background:**

The albumin–bilirubin (ALBI) score, originally developed to assess hepatic reserve, has shown prognostic value in liver and systemic diseases. Whether the ALBI score is associated with diabetic kidney disease (DKD) progression remains unclear.

**Methods:**

We conducted a retrospective single-centre study including 113 patients with DKD hospitalized between January 2016 and May 2023. The ALBI score was calculated from serum albumin and total bilirubin. Patients were followed for 2–7 years, and DKD progression was evaluated by annual percentage change in estimated glomerular filtration rate (eGFR). Associations between the ALBI score quartiles and renal outcomes were assessed using correlation analyses, logistic regression, and receiver operating characteristic (ROC) curves.

**Results:**

A total of 113 patients with DKD were included in the analysis. Higher ALBI score was associated with a more rapid annual decline in eGFR and greater urinary protein excretion. Across ALBI quartiles, the annual percentage change in eGFR differed significantly, and 24-h urine total protein quantification (UTP) increased progressively with increasing quartiles. Correlation analysis showed that the ALBI score was positively correlated with UTP and negatively correlated with annual percentage change in eGFR. ROC analysis suggested an exploratory cut-off value of −2.2350 (area under the curve (AUC) = 0.656, *P* = 0.004) for distinguishing patients with rapid eGFR decline, although the discriminatory performance was modest, and this threshold has not been clinically validated. After multivariable adjustment, higher ALBI score quartiles remained significantly associated with rapid eGFR decline (*P* for trend = 0.024). In the adjusted Model 3, compared with Quartile 1, only Quartile 4 remained significantly associated with rapid eGFR decline, whereas Quartiles 2 and 3 did not reach statistical significance.

**Conclusions:**

This study suggests that the ALBI score was associated with DKD progression and may serve as a potential supplementary indicator for identifying patients at higher risk of rapid eGFR decline.

## Introduction

Diabetic kidney disease (DKD), a common microvascular complication of diabetes, is a leading cause of chronic kidney disease (CKD) and end-stage renal disease (ESRD) worldwide (Fu et al., 2019; Li et al., 2021). Its clinical course is marked by progressive renal functional decline, with the estimated glomerular filtration rate (eGFR) serving as a central metric for monitoring disease trajectory and forecasting outcomes (Levey, Inker & Coresh, 2014). Metabolic dysregulation has emerged as a major driver of DKD progression and is also implicated in the pathogenesis of liver disease. Among liver manifestations of metabolic dysfunction, metabolic dysfunction-associated steatotic liver disease (MASLD), the current nomenclature for metabolically driven steatotic liver disease, has been increasingly recognized as closely associated with CKD, and affected individuals appear to have a substantially higher risk of incident renal dysfunction (Bilson et al., 2024). Recent evidence indicates a strong link between liver fibrosis and DKD, giving rise to the concept of a “cardio–renal–hepatic–metabolic axis,” wherein worsening liver fibrosis may parallel renal deterioration (Benlloch, Moncho & Górriz, 2024).

The albumin–bilirubin (ALBI) score, initially proposed as a straightforward index of hepatic function, has been validated extensively in liver fibrosis and cirrhosis (Bozkurt et al., 2016; Tian et al., 2024). Beyond hepatology, the ALBI score has shown prognostic value in systemic disorders such as acute heart failure and idiopathic dilated cardiomyopathy (Jiang et al., 2022; Matsue et al., 2020). Whether the ALBI score can similarly predict DKD progression, however, remains unclear. Here we examined the association between the ALBI score and DKD progression, with the goal of informing precision management and early intervention strategies in DKD.

## Materials and Methods

### Subjects and data

This retrospective study was approved by the Ethics Committee of Dongzhimen Hospital, Beijing University of Chinese Medicine (Approval No. 2023DZMEC-134-01). As all data were extracted from electronic medical records of hospitalized patients, the requirement for informed consent was waived. The study design and data collection procedures were based on our previously published work (Cao et al., 2024), with modifications in the inclusion period and clinical variables. Specifically, individuals aged ≥18 years who were diagnosed with DKD according to the Chinese Guidelines for the Diagnosis and Treatment of DKD (Expert Group of Chinese Society of Nephrology, 2021) between January 2016 and May 2023 at Dongzhimen Hospital were screened for inclusion. Patients were excluded if they had incomplete clinical data, a history of renal surgery, infection, malignant tumor, documented acute kidney injury (AKI) at baseline or during follow-up (based on diagnostic codes), acute diabetic complications within the preceding month (including diabetic ketoacidosis and hyperosmolar hyperglycemic state/coma). Patients with severe liver disease were excluded, including those with decompensated liver disease, liver failure, or severe hepatic impairment equivalent to Child–Pugh class C. Those who had been hospitalized in the nephrology department at least twice during the observation period of 2–7 years were included in the analysis. In routine clinical practice, these admissions were mainly for chronic disease management, such as poor glycemic control, progression of renal dysfunction, worsening edema, or related conditions, rather than for identifying episodes of acute kidney injury. The ALBI score and other clinical and laboratory variables used in the analysis were obtained from the first hospitalization, whereas subsequent follow-up admissions were used only to calculate changes in eGFR.

### Laboratory assays

Laboratory assessments were performed following procedures described in our previous study (Cao et al., 2024), with additional measurements of serum albumin incorporated in the current analysis. Venous blood samples were collected after an overnight fast of at least 8 h. Biochemical parameters, including glycated hemoglobin A1c (HbA1c, %), blood glucose (GLU, mmol/L), serum creatinine (Scr, µmol/L), blood urea nitrogen (BUN, mmol/L), uric acid (UA, µmol/L), alanine aminotransferase (ALT, U/L), aspartate aminotransferase (AST, U/L), gamma-glutamyl transpeptidase (GGT, U/L), total bilirubin (TBIL, µmol/L), direct bilirubin (DBIL, µmol/L), indirect bilirubin (IBIL, µmol/L), 24-h urine total protein quantification (UTP, g/24 h) and serum albumin (ALB, g/L), were measured using an automated biochemical analyzer (Beckman DXC-800; Beckman, Brea, CA, USA). The eGFR was calculated using the CKD-Epidemiology Collaboration (EPI) equation (Levey et al., 2009). Body mass index (BMI) was used to assess obesity, which was defined as BMI >28 kg/m^2^ according to guideline criteria (National Clinical Practice Guideline on Obesity Management Editorial Committee, 2025).

### Statistical analysis

Continuous variables with normal distribution were expressed as mean ± standard deviation (SD) and compared using the independent-samples t-test. Non-normally distributed variables (Kolmogorov–Smirnov test, *P* < 0.1) were summarized as median (interquartile range, IQR) and compared using the Mann–Whitney U test. Categorical variables were analyzed with the $\chi^2$ test. For comparisons among ALBI score quartiles, one-way analysis of variance (ANOVA) or the Kruskal–Wallis test was applied, as appropriate. Binary logistic regression analysis was used to examine the association between ALBI score quartiles and rapid eGFR decline, with Quartile 1 as the reference group. Model 1 was unadjusted. Model 2 was adjusted for obesity, hypertension (HT), hyperlipidemia, coronary heart disease (CHD), cerebrovascular disease (CVD), smoking, alcohol consumption, renin-angiotensin system inhibitor (RASI), sex, age, and diabetes duration. Model 3 was further adjusted for UTP, BUN, total protein (TP), ALT, AST, GLU, and HbA1c based on Model 2. Correlations between the ALBI score and clinical indicators were assessed using partial correlation analysis after adjustment for potential confounding variables. Receiver operating characteristic (ROC) curve analysis was performed to evaluate the discriminatory ability of the ALBI score for rapid decline in eGFR, and the optimal cutoff value was determined according to the maximum Youden index. All analyses were performed with SPSS version 25.0 (IBM, Chicago, IL, USA), and a two-sided *P* < 0.05 was considered statistically significant. Correlation plots were generated using GraphPad Prism version 9.5.

### Definition of ALBI score and renal function decline

ALBI score was calculated as follows:

ALBI score = (log_10_ bilirubin [µmol/L] × 0.66) + (albumin [g/L] × –0.085) (Johnson et al., 2015).

The rate of DKD progression was evaluated by the annual percentage change in eGFR over time, calculated using the baseline and final available eGFR values during follow-up. The annual percentage change in eGFR was derived as [(final eGFR – baseline eGFR)/baseline eGFR/follow-up years] × 100. Based on prior literature (Kovačević et al., 2022), an eGFR slope of –5.48% per year was selected as the threshold for renal function decline. Because a negative value indicates a decline in eGFR, patients were further classified according to the magnitude of eGFR decline:

Non-rapid decline group: eGFR decline <5.48%/year.

Rapid decline group: eGFR decline ≥5.48%/year.

## Results

### General data and correlation analysis

A total of 113 patients with DKD were included in the baseline analysis. According to the annual percentage change in eGFR, 55 patients were assigned to non-rapid decline group (eGFR decline <5.48%/year) and 58 patients were assigned to rapid decline group (eGFR decline ≥5.48%/year). The two groups were generally comparable at baseline with respect to sex, age, monitoring time, duration of diabetes, hypertension, hyperlipidemia, coronary heart disease, cerebrovascular disease, alcohol consumption, DPP4 inhibitor use, obesity, HbA1c, glucose, Scr, eGFR, globulin (GLB), TBIL, IBIL, ALT, GGT, and ALP (all *P* > 0.05). Compared with the rapid decline group, the non-rapid decline group had significantly lower proportions of smokers and RASI users, significantly lower UTP and BUN levels, and significantly higher TP, ALB, DBIL, and AST levels. In addition, the mean ALBI score was significantly lower in non-rapid decline group than in rapid decline group (−2.38 ± 0.29 *vs*. −2.19 ± 0.05, *P* = 0.002) (Table 1).

**Table 1:** Baseline demographic and clinical characteristics of the two groups.

	Non-rapid decline group eGFR decline <5.48%/year	Rapid decline group eGFR decline ≥5.48%/year	z/t/ $\chi^2$ value	*P* value
Case	55	58		
Gender, males/females	35/20	42/16	1.002	0.317
Age, years	58.31 ± 11.31	54.86 ± 10.70	1.665	0.099
Monitoring time, years	3.93 ± 1.40	3.68 ± 1.26	1.015	0.313
Diabetes duration, years	11.53 ± 6.28	12.58 ± 6.81	−0.857	0.394
HT (%)	38 (69.1)	40 (69.0)	0.001	0.989
Hyperlipidemia	32 (58.2)	39 (67.2)	0.992	0.319
CHD (%)	19 (34.5)	16 (27.6)	0.639	0.424
CVD (%)	17 (30.9)	16 (27.6)	0.151	0.698
Smoking (%)	28 (50.9)	41 (70.7)	4.646	0.031
Alcohol consumption (%)	25 (45.4)	20 (34.5)	1.418	0.234
RASI (%)	13 (23.6)	28 (48.3)	7.413	0.006
DPP4 (%)	1 (1.8)	3 (5.2)	0.930	0.335
Obesity (%)	24 (43.6)	17 (29.3)	2.506	0.113
HbA1c (%)	8.40 (2.37)	8.29 (2.68)	−0.164	0.870
GLU (mmol/L)	9.80 (5.25)	10.20 (6.80)	−0.741	0.459
UTP (g/24 h)	0.20 (0.54)	0.84 (1.83)	−3.804	<0.001
Scr (μmol/L)	66.73 ± 16.47	72.36 ± 20.85	−1.589	0.115
eGFR (ml/min)	97.08 ± 19.21	95.27 ± 19.44	0.497	0.620
BUN (mmol/L)	4.70 (1.95)	5.50 (2.10)	−2.316	0.021
TP (g/L)	64.54 ± 4.87	61.52 ± 6.07	2.915	0.004
ALB (g/L)	36.59 ± 3.64	34.08 ± 4.16	3.411	0.001
GLB (g/L)	27.95 ± 4.18	27.44 ± 3.90	0.675	0.501
TBIL (μmol/L)	12.30 (7.10)	11.65 (5.70)	−0.755	0.450
DBIL (μmol/L)	3.61 ± 1.76	2.91 ± 1.40	2.330	0.022
IBIL (μmol/L)	9.70 (4.15)	9.30 (4.20)	−0.394	0.694
ALT (U/L)	19.00 (12.50)	16.50 (12.00)	−1.757	0.079
AST (U/L)	19.00 (10.50)	18.00 (7.00)	−2.154	0.031
GGT (U/L)	26.00 (26.00)	23.50 (17.00)	−0.586	0.558
ALP (U/L)	66.00 (23.50)	63.00 (27.00)	−1.052	0.293
ALBI score	−2.38 ± 0.29	−2.19 ± 0.36	−3.143	0.002

**Notes:**

Values are expressed as mean ± SD, median (IQR), or *n* (%).

Abbreviations: eGFR, estimated glomerular filtration rate; HT, hypertension; CHD, coronary heart disease; CVD, cerebrovascular disease; RASI, renin-angiotensin system inhibitor; DPP4, dipeptidyl peptidase-4 inhibitor; HbA1c, glycated hemoglobin A1c; GLU, blood glucose; UTP, urinary total protein; Scr, serum creatinine; BUN, blood urea nitrogen; TP, total protein; ALB, albumin; GLB, globulin; TBIL, total bilirubin; DBIL, direct bilirubin; IBIL, indirect bilirubin; ALT, alanine aminotransferase; AST, aspartate aminotransferase; GGT, gamma-glutamyl transferase; ALP, alkaline phosphatase; ALBI score, albumin-bilirubin score.

When patients were stratified according to ALBI score quartiles, UTP increased significantly across quartiles (*P* = 0.009). The annual percentage change in eGFR also differed significantly among quartiles, with median values of −1.79, −6.65, −6.56, and −11.15 from Quartile 1 to Quartile 4, respectively (*P* = 0.022). However, no significant differences were observed in Scr, BUN, or eGFR among the four quartile groups (Table 2).

**Table 2:** Association between ALBI score quartiles and renal function.

Variable	Quartile 1	Quartile 2	Quartile 3	Quartile 4	*z/F* value	*P* value
Cases, *n*	30	25	30	28		
UTP (g/24 h)	0.19 (0.60)	0.42 (0.67)	0.57 (0.62)	1.34 (1.90)	11.457	0.009
Scr (μmol/L)	70.70 ± 18.40	66.64 ± 20.89	66.30 ± 19.24	74.68 ± 17.20	1.214	0.308
BUN (mmol/L)	5.00 (1.80)	4.80 (2.10)	5.00 (2.10)	5.55 (2.55)	2.674	0.445
eGFR (ml/min)	96.43 ± 21.26	96.82 ± 21.46	99.12 ± 18.90	92.08 ± 15.27	0.662	0.577
Annual percentage in eGFR (%)	−1.79 (−5.4)	−6.65 (−14.11)	−6.56 (−15.59)	−11.15 (−14.4)	9.635	0.022

**Notes:**

Data are presented as mean ± SD, median (IQR), or *n*, as appropriate. Quartile 1–4 indicate the first to fourth quartile of ALBI score. Comparisons among the four groups were performed using one-way ANOVA or the Kruskal–Wallis test, as appropriate.

Abbreviations: ALBI score, albumin-bilirubin score; UTP, urinary total protein; Scr, serum creatinine; BUN, blood urea nitrogen; eGFR, estimated glomerular filtration rate.

**Figure 1:**
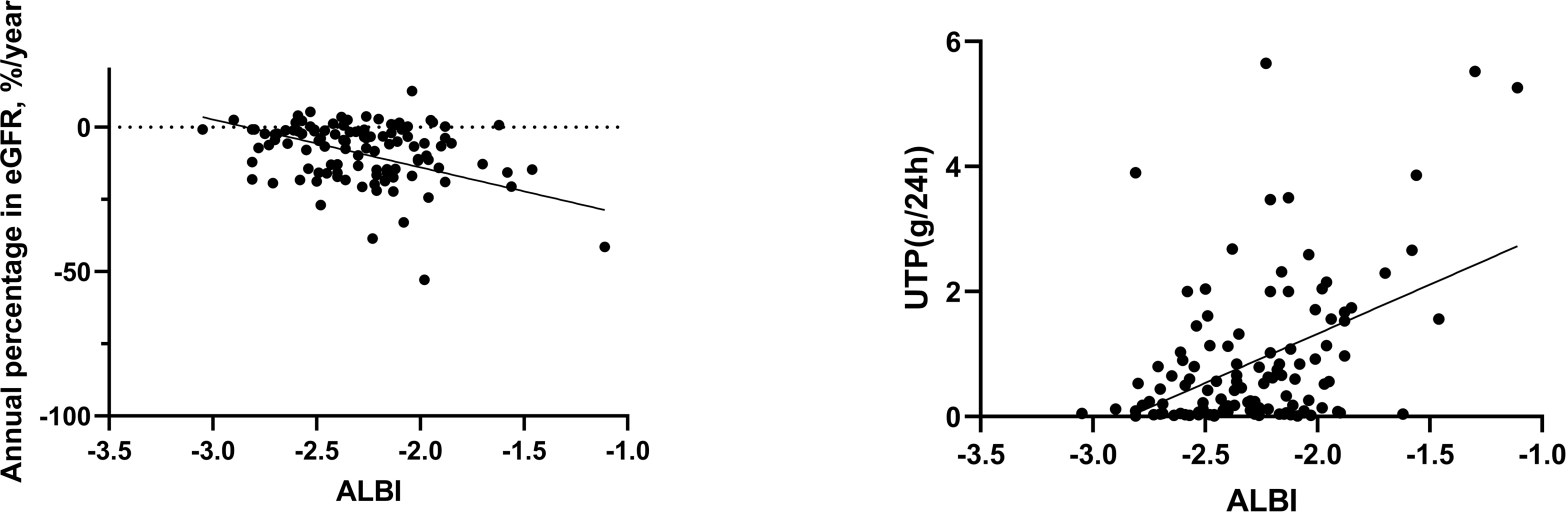
Correlation between ALBI score and UTP, annual percentage in eGFR.

Correlation analysis showed that the ALBI score was significantly positively correlated with UTP (r/rs = 0.442, *P* < 0.001) and significantly negatively correlated with annual percentage change in eGFR (r/rs = −0.412, *P* < 0.001). No significant correlations were found between the ALBI score and Scr, eGFR, GLU, HbA1c, ALT, or AST (Table 3, Fig. 1). Further correlation analysis showed that annual percentage change in eGFR was significantly negatively correlated with UTP (*P* < 0.001), and significantly positively correlated with TP (*P* = 0.001), and ALB (*P* = 0.008). No significant correlations were observed between annual percentage change in eGFR and TBIL, DBIL, IBIL, ALT, GLU, or HbA1c (Table 4).

**Table 3:** Correlations between the ALBI score and clinical indicators.

Variable	*r/r*_*s*_ value	*P* value
UTP (g/24 h)	0.442	<0.001
Scr (μmol/L)	0.104	0.288
eGFR (ml/min)	−0.081	0.408
GLU (mmol/L)	−0.024	0.809
HbA1c (%)	0.009	0.928
ALT (U/L)	−0.043	0.664
AST (U/L)	−0.054	0.580
Annual percentage in eGFR (%)	−0.412	<0.001

**Notes:**

Correlation coefficients are presented as r or r_s_, as appropriate. Pearson correlation analysis was used for normally distributed variables, and Spearman rank correlation analysis was used for non-normally distributed variables.

Abbreviations: UTP, urinary total protein; Scr, serum creatinine; eGFR, estimated glomerular filtration rate; GLU, blood glucose; HbA1c, glycated hemoglobin A1c; ALT, alanine aminotransferase; AST, aspartate aminotransferase.

**Table 4:** Correlations between the annual percentage in eGFR and clinical indicators.

Variable	*r*_*s*_ value	*P* value
UTP	−0.454	<0.001
TP	0.299	0.001
ALB	0.249	0.008
TBIL	0.033	0.730
DBIL	0.152	0.109
IBIL	0.002	0.981
ALT	0.178	0.060
AST	0.206	0.029
GLU	−0.066	0.486
HbA1c	−0.006	0.954

**Notes:**

Correlation coefficients are presented as rs. Spearman rank correlation analysis was used for non-normally distributed variables.

Abbreviations: UTP, urinary total protein; TP, total protein; ALB, albumin; TBIL, total bilirubin; DBIL, direct bilirubin; IBIL, indirect bilirubin; ALT, alanine aminotransferase; AST, aspartate aminotransferase; GLU, blood glucose; HbA1c, glycated hemoglobin A1c; eGFR, estimated glomerular filtration rate.

**Figure 2:**
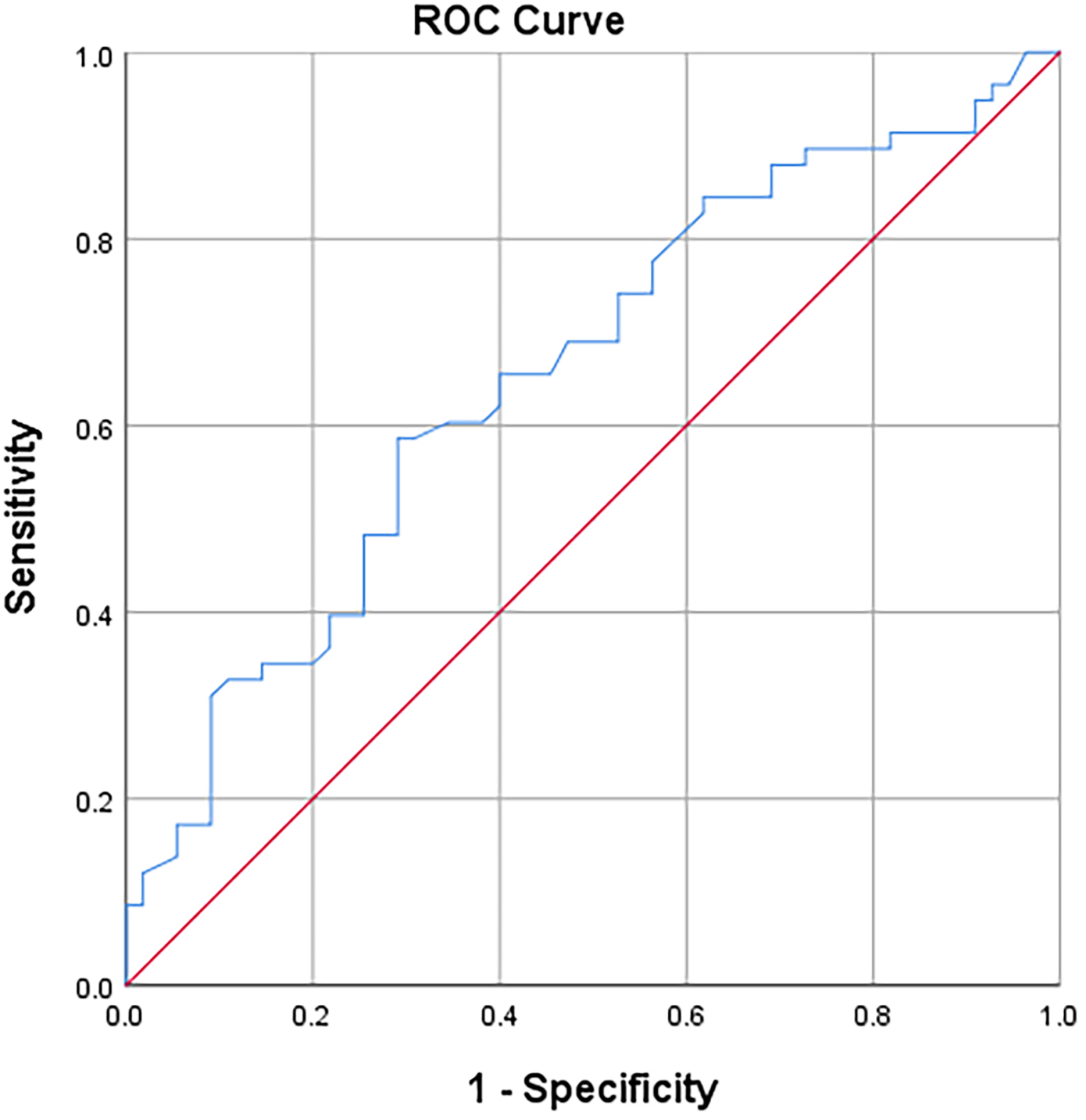
Receiver operating characteristic (ROC) curve analysis of the ALBI score. The area under the ROC curve (AUC) was 0.656 (95% CI [0.556–0.757], *P* = 0.004). The optimal cutoff value was −2.2350, yielding a sensitivity of 58.6% and a specificity of 70.0%.

Receiver operating characteristic analysis showed that the ALBI score had a modest ability to identify rapid eGFR decline, with an area under the curve (AUC) of 0.656 (95% confidence interval (CI) [0.556–0.757], *P* = 0.004). The exploratory cutoff value was −2.2350, with a sensitivity of 58.6% and a specificity of 70.0% (Fig. 2).

The proportions of patients with rapid eGFR decline increased across ALBI score quartiles, from 30.0% in Quartile 1 to 52.0%, 53.3%, and 71.4% in Quartiles 2, 3, and 4, respectively. In the unadjusted model, compared with Quartile 1, the odds ratios (ORs) for rapid eGFR decline were 2.528 (95% CI [0.836–7.747]), 2.667 (95% CI [0.924–7.699]), and 5.833 (95% CI [1.880–18.099]) for Quartiles 2, 3, and 4, respectively, with a significant trend across quartiles (*P* for trend = 0.003). After adjustment, the corresponding ORs were 2.354 (95% CI [0.600–9.241]), 2.829 (95% CI [0.700–11.437]), and 10.538 (95% CI [2.398–46.318]) in Model 2, and 3.507 (95% CI [0.738–16.662]), 4.590 (95% CI [0.686–30.729]), and 11.187 (95% CI [1.449–86.343]) in Model 3. The trend remained significant in both adjusted models (*P* for trend = 0.002 and 0.024, respectively) (Table 5). In the adjusted Model 3, compared with Quartile 1, only Quartile 4 remained significantly associated with rapid eGFR decline, whereas Quartiles 2 and 3 did not reach statistical significance.

**Table 5:** Association between ALBI score quartiles and rapid decline in eGFR.

	Quartiles of variables				
Variable	Quartile 1	Quartile 2	Quartile 3	Quartile 4	*P* for trend
ALBI score	~−2.51	−2.51~−2.28	−2.28~−2.09	−2.09~	
Events, %	30%	52.0%	53.3%	71.4%	
Model 1	1	2.528 (0.836~7.747)	2.667 (0.924~7.699)	5.833 (1.880~18.099)	0.003
*P* value	–	0.101	0.070	0.002	
Model 2	1	2.354 (0.600~9.241)	2.829 (0.700~11.437)	10.538 (2.398~46.318)	0.002
*P* value	–	0.220	0.145	0.002	
Model 3	1	3.507 (0.738~16.662)	4.590 (0.686~30.729)	11.187 (1.449~86.343)	0.024
*P* value	–	0.115	0.116	0.021	

**Notes:**

Model 1, unadjusted.

Model 2, adjusted for obesity, HT, hyperlipidemia, CHD, CVD, smoking, alcohol consumption, RASI, sex, age, and diabetes duration.

Model 3, further adjusted for UTP, BUN, TP, ALT, AST, GLU, and HbA1c based on Model 2.

## Discussion

This study examined the association between the ALBI score and the progression of DKD. We found that a higher ALBI score was associated with a more rapid annual decline in eGFR, suggesting that the ALBI score may have modest prognostic value in identifying patients at increased risk of renal function decline. Our previous work demonstrated that sustained elevations of serum bilirubin within the physiological range attenuate DKD progression, likely through its antioxidant and anti-inflammatory effects (Cao et al., 2024), while other studies have established a close relationship between serum albumin and both the onset and progression of DKD (Wang et al., 2025). By integrating these two components, the ALBI score captures nutritional status, inflammation and oxidative stress in a single index. Here we demonstrate that the ALBI score correlates positively with DKD progression and that higher ALBI score quartiles are associated with faster eGFR decline. In the fully adjusted model, only Quartile 4 remained statistically significant *vs*. Quartile 1, while Quartiles 2 and 3 did not. These findings suggest that the ALBI score may serve as a simple supplementary indicator for risk assessment in DKD.

Liver and kidney diseases share overlapping pathophysiological pathways, and growing evidence points to a bidirectional interplay between the two organs during disease progression. Benlloch, Moncho & Górriz (2024) highlighted the shared pathological basis of cardiovascular disease, type 2 diabetes, chronic kidney disease, and non-alcoholic fatty liver disease, introducing the concept of a “cardio–renal–hepatic–metabolic axis” as a central determinant of systemic disease development. A 5-year retrospective cohort study reported that patients with metabolic abnormalities and fatty liver disease exhibited a higher incidence of CKD (Kwon et al., 2023). The ALBI score was originally introduced as a simple metric for assessing hepatic functional reserve and prognosis in hepatocellular carcinoma (Johnson et al., 2015). Since then, its validity has been confirmed across international multicentre cohorts, and its use has expanded to a broad spectrum of chronic liver diseases, including both compensated and decompensated cirrhosis (Toyoda & Johnson, 2022; Navadurong et al., 2023) and metabolic-associated fatty liver disease (MAFLD) (Zhou et al., 2025). Beyond hepatology, the ALBI score has shown prognostic value in diverse conditions such as atrial fibrillation, pancreatic cancer, and variceal (Zhang et al., 2025; Azili et al., 2023; Inoue-Yuri et al., 2022). In nephrology, emerging evidence implicates the ALBI score in acute kidney injury (AKI). It has been reported to predict contrast-induced AKI and long-term mortality after elective percutaneous coronary intervention (PCI) (Chen et al., 2025), while analyses from the MIMIC-IV critical care database indicate that higher ALBI score grades correlate with poorer outcomes in critically ill patients with AKI (Yang et al., 2024).

Together with previous evidence, our findings indicate that the ALBI score reflects not only hepatic functional reserve and fibrosis severity but also renal risk. To our knowledge, few studies have examined the association between the ALBI score and eGFR decline in patients with DKD. Our findings provide preliminary evidence that higher ALBI score is associated with faster renal function decline. This observation underscores the interconnectedness of liver and kidney function in metabolic disease and offers new opportunities for risk stratification and targeted intervention.

In the present study, RASI use was more frequent in the rapid eGFR decline group. It may reflect confounding by indication, because RASI therapy is commonly prescribed to patients with hypertension, albuminuria/proteinuria, or greater baseline renal risk. Therefore, patients receiving RASI may have had more advanced renal involvement at baseline. Although RASI use was included in the adjusted model and UTP was further adjusted for in Model 3, residual confounding could not be fully excluded. In particular, the retrospective design did not allow us to comprehensively account for RASI dose, treatment duration, adherence, changes during follow-up, or the specific clinical indications for RASI prescription. Moreover, because RASI therapy may reduce proteinuria and slow renal function decline, incomplete characterization of medication exposure may have biased the observed associations in either direction. These issues should be addressed in future prospective studies with detailed longitudinal medication data.

This study has several limitations. It was a single-centre retrospective analysis with a relatively small sample size, which may have introduced selection bias and limits the generalizability of the findings. Although the multivariable models adjusted for several clinical factors, including UTP and RASI use in Model 3, residual confounding cannot be fully excluded. In particular, because the ALBI score includes serum albumin, it may be influenced by baseline proteinuria, inflammation, and nutritional status. The marked difference in UTP between groups indicates that proteinuria-related confounding may remain even after statistical adjustment. Although documented AKI was excluded based on diagnostic codes, undocumented AKI or acute illness-related creatinine fluctuations may still have influenced the calculated annual eGFR change. In addition, causes of hospitalization and treatment changes during follow-up, including initiation, discontinuation, or dose adjustment of nephroprotective therapies, were not fully captured and may have influenced eGFR decline, thereby contributing to residual confounding. Moreover, longitudinal changes in the ALBI score were not assessed, and mechanistic investigations were not undertaken, constraining deeper insights into its role in DKD progression. Validation in larger, multicentre prospective cohorts and mechanistic studies will be essential to substantiate and extend these observations.

## Conclusion

This study evaluated the association between the ALBI score and DKD progression. We found that higher ALBI score was associated with faster annual eGFR decline, suggesting that it may have modest prognostic value for renal function decline in DKD. However, the proposed cutoff value should be considered exploratory and requires external validation before clinical use. Building on prior evidence of the protective effects of bilirubin and the detrimental impact of hypoalbuminaemia, the ALBI score integrates these two parameters into a single index that captures systemic inflammation, oxidative stress, and nutritional status, offering a more comprehensive measure of disease risk.

## Supplemental Information

10.7717/peerj.21648/supp-1 Supplemental Information 1 Raw data.

10.7717/peerj.21648/supp-2 Supplemental Information 2 STROBE Statement.

## Additional Information and Declarations

### Data Availability

The following information was supplied regarding data availability:

The raw measurements are available in the Supplemental Files.
