# Supplementary material for: The albumin–bilirubin score is associated with renal decline in diabetic kidney disease: a retrospective study
Source: PeerJ. 2026 Aug 14;14:e21648. doi: 10.7717/peerj.21648 (PMC13480139; doi:10.7717/peerj.21648)
Supplement: Supplementary file 1 [file peerj-14-21648-s002.doc]

STROBE Statement—Checklist of items that should be included in reports of ***cross-sectional studies***

|  | Item No | Recommendation |
| --- | --- | --- |
| **Title and abstract**  YES | 1 | (*a*) Indicate the study’s design with a commonly used term in the title or the abstract Line 13 |
|  |  | (*b*) Provide in the abstract an informative and balanced summary of what was done and what was found Line 11-25 |
| Introduction | | |
| Background/rationale YES | 2 | Explain the scientific background and rationale for the investigation being reported  Line 30-37 |
| Objectives YES | 3 | State specific objectives, including any prespecified hypotheses Line 38-44 |
| Methods | | |
| Study design YES | 4 | Present key elements of study design early in the paper Line 47 |
| Setting YES | 5 | Describe the setting, locations, and relevant dates, including periods of recruitment, exposure, follow-up, and data collection Line 48-58 |
| Participants YES | 6 | (*a*) Give the eligibility criteria, and the sources and methods of selection of participants Line 50-56 |
| Variables YES | 7 | Clearly define all outcomes, exposures, predictors, potential confounders, and effect modifiers. Give diagnostic criteria, if applicable Line 83-92 |
| Data sources/ measurement YES | 8* | For each variable of interest, give sources of data and details of methods of assessment (measurement). Describe comparability of assessment methods if there is more than one group Line 83-92 |
| Bias NO | 9 | Describe any efforts to address potential sources of bias NO |
| Study size | 10 | Explain how the study size was arrived at NO |
| Quantitative variables YES | 11 | Explain how quantitative variables were handled in the analyses. If applicable, describe which groupings were chosen and why Line 91-92 |
| Statistical methods YES | 12 | (*a*) Describe all statistical methods, including those used to control for confounding Line 72-81 |
|  |  | (*b*) Describe any methods used to examine subgroups and interactions No applicable |
|  |  | (*c*) Explain how missing data were addressed No applicable |
|  |  | (*d*) If applicable, describe analytical methods taking account of sampling strategy No applicable |
|  |  | (*e*) Describe any sensitivity analyses No applicable |
| Results | | |
| Participants YES | 13* | (a) Report numbers of individuals at each stage of study—eg numbers potentially eligible, examined for eligibility, confirmed eligible, included in the study, completing follow-up, and analysed Line 96 |
|  |  | (b) Give reasons for non-participation at each stage |
|  |  | (c) Consider use of a flow diagram |
| Descriptive data YES | 14* | (a) Give characteristics of study participants (eg demographic, clinical, social) and information on exposures and potential confounders Line 97-103 |
|  |  | (b) Indicate number of participants with missing data for each variable of interest No applicable |
| Outcome data YES | 15* | Report numbers of outcome events or summary measures Line 102-103 |
| Main results YES | 16 | (*a*) Give unadjusted estimates and, if applicable, confounder-adjusted estimates and their precision (eg, 95% confidence interval). Make clear which confounders were adjusted for and why they were included Line 105-123 |
|  |  | (*b*) Report category boundaries when continuous variables were categorized Line 106 |
|  |  | (*c*) If relevant, consider translating estimates of relative risk into absolute risk for a meaningful time period Line 105-123 |
| Other analyses YES | 17 | Report other analyses done—eg analyses of subgroups and interactions, and sensitivity analyses No applicable |
| Discussion | | |
| Key results YES | 18 | Summarise key results with reference to study objectives Line 126-133 |
| Limitations YES | 19 | Discuss limitations of the study, taking into account sources of potential bias or imprecision. Discuss both direction and magnitude of any potential bias Line 178-184 |
| Interpretation YES | 20 | Give a cautious overall interpretation of results considering objectives, limitations, multiplicity of analyses, results from similar studies, and other relevant evidence Line 138-168 |
| Generalisability YES | 21 | Discuss the generalisability (external validity) of the study results Line 170-177 |
| Other information | | |
| Funding YES | 22 | Give the source of funding and the role of the funders for the present study and, if applicable, for the original study on which the present article is based Line 204-207 |

*Give information separately for exposed and unexposed groups.

**Note:** An Explanation and Elaboration article discusses each checklist item and gives methodological background and published examples of transparent reporting. The STROBE checklist is best used in conjunction with this article (freely available on the Web sites of PLoS Medicine at http://www.plosmedicine.org/, Annals of Internal Medicine at http://www.annals.org/, and Epidemiology at http://www.epidem.com/). Information on the STROBE Initiative is available at www.strobe-statement.org.
